# Supplementary material for: Materials analysis and focused ion beam nanofabrication of topological insulator Bi2Se3
Source: Sci Rep. 2017 Oct 18;7:13466. doi: 10.1038/s41598-017-13863-6 (PMC5647386; doi:10.1038/s41598-017-13863-6)
Supplement: Supplementary file 1 — Supplementary Information [file 41598_2017_13863_MOESM1_ESM.pdf]

Supporting Information

Materials analysis and focused ion beam nanofabrication of topological insulator Bi_2Se_3

Sarah Friedensen[1‡], *Jerome Mlack*[1‡], *Marija Drndić*[1] *

[1] Department of Physics and Astronomy, University of Pennsylvania,

Philadelphia, Pennsylvania 19104, United States

[‡]These authors have contributed equally.

[*]Corresponding author: Marija Drndić, drndic@physics.upenn.edu

Table of Contents:

1. **Diagrams of FIB effects on Bi_2Se_3**
2. **Additional images and analysis of FIB-thinned TEM sample**
3. **Elemental analysis of wire sample**
4. **Diagram of sample position in relation to TEM beam**
5. **SAED of silicon nitride membrane**

Section 1: Diagrams of FIB effects on Bi_2Se_3

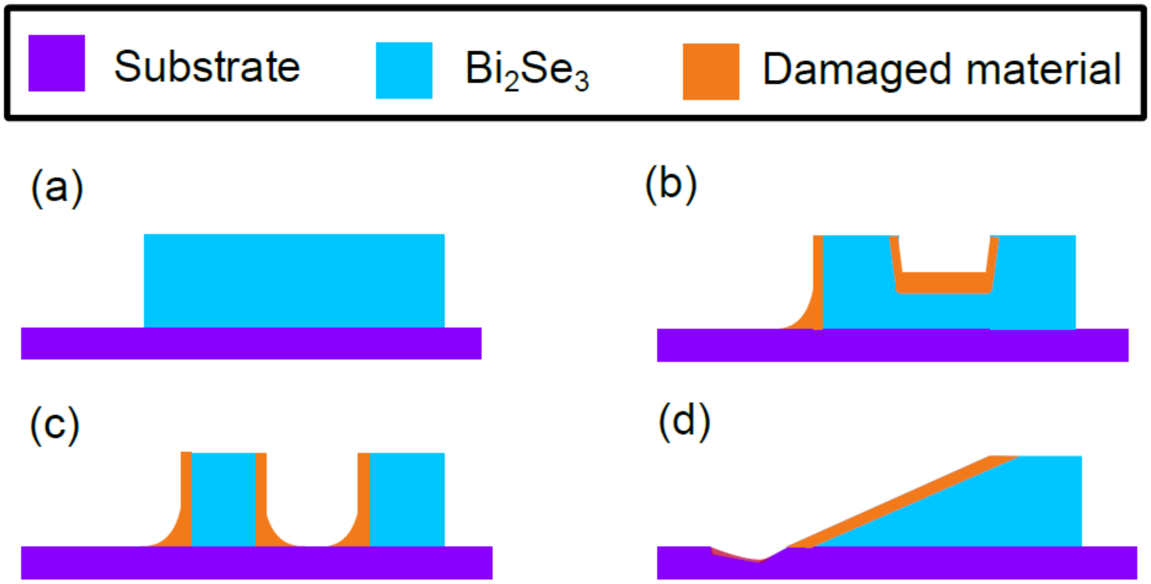

Figure SI 1.1: Diagram of the material effects that the FIB has on Bi_2Se_3 when thinned, cut directly, and cut at an angle. (a) Uncut Bi_2Se_3 (teal) on substrate (purple). (b) Bi_2Se_3 which has been milled to the substrate on the left and thinned in the center. The sidewalls are damaged from the fact that the beam is not perfectly collimated, with some damaged material (orange) remaining at the base of the fully-milled region and some covering the entire thinned region. (c) Sample milled on the left and fully milled in the middle. (d) Angle-milled sample, showing damaged covering (orange) from beam collimation and unsputtered material.

Section 2: Additional images and analysis of FIB thinned TEM sample

.

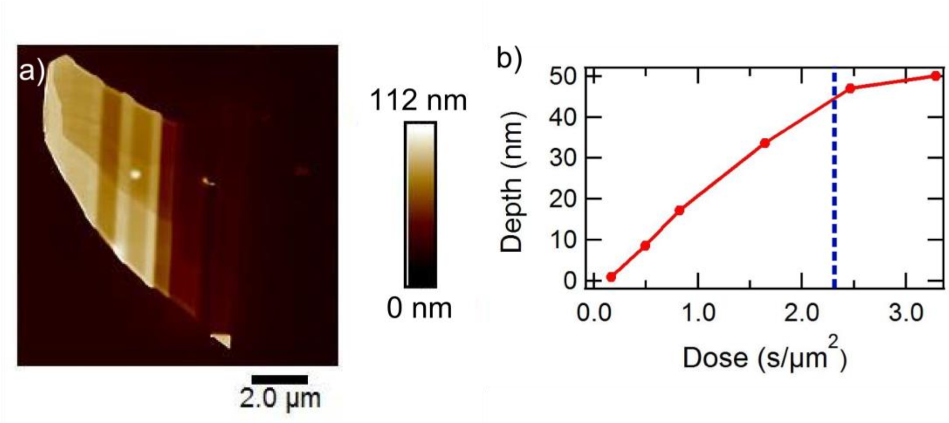

Figure SI 2.1: Dose versus mill depth from AFM map of thinned sample imaged in TEM. (a) AFM image of FIB-thinned sample. (b) Dose versus mill depth, dashed blue line represents approximate dose at which the beam milled through the Bi_2Se_3 and began milling the silicon nitride.

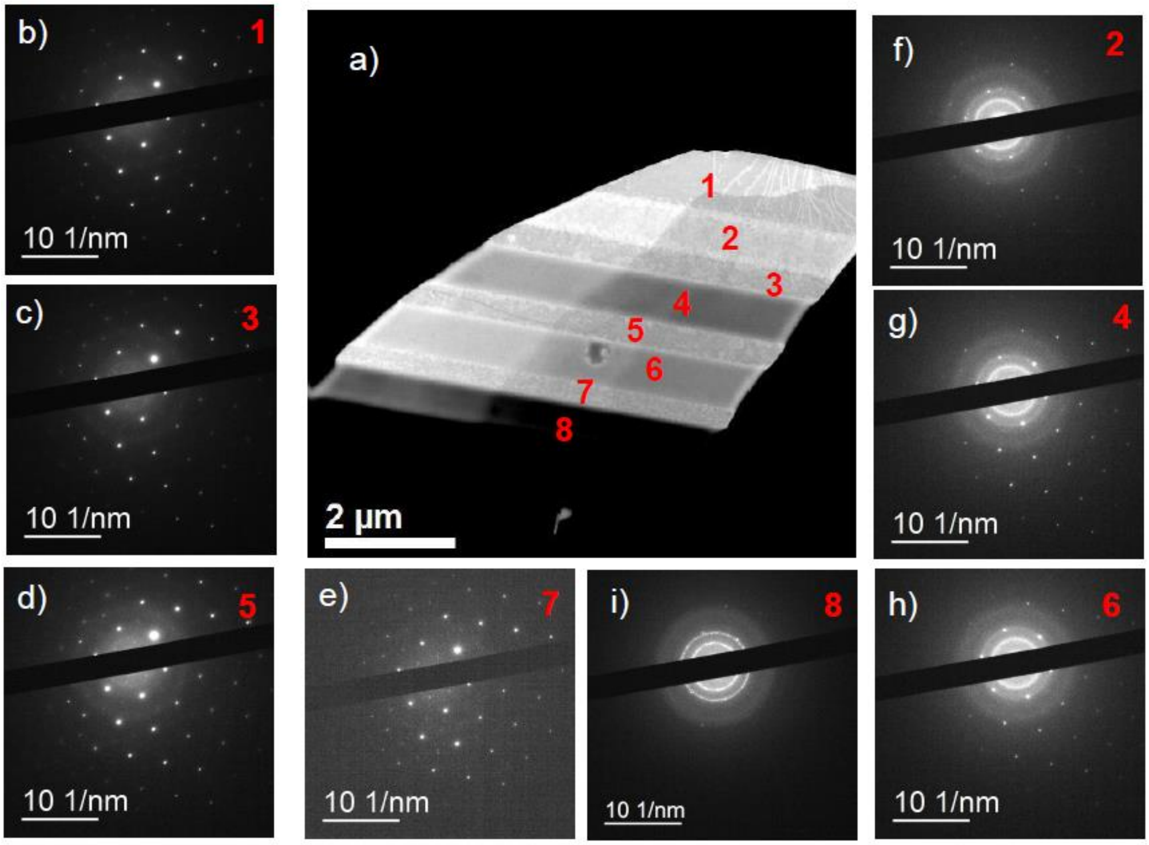

Figure SI 2.2: Counts per second image and diffractions images of FIB thinned Bi_2Se_3 flake. (a) Counts per second image of FIB thinned Bi_2Se_3 flake. Red numbers correspond to the diffraction images shown in (b-h). (b) Diffraction pattern from region 1 of (a). (c) Diffraction pattern from region 3 of (a). (d) Diffraction pattern from region 5 of (a). (e) Diffraction pattern from region 7 of (a). (f) Diffraction pattern from region 2 of (a). (g) Diffraction pattern from region 4 of (a). (h) Diffraction pattern from region 6 of (a). (i) Diffraction pattern from region 8 of (a).

Section 3: Elemental analysis of wire sample

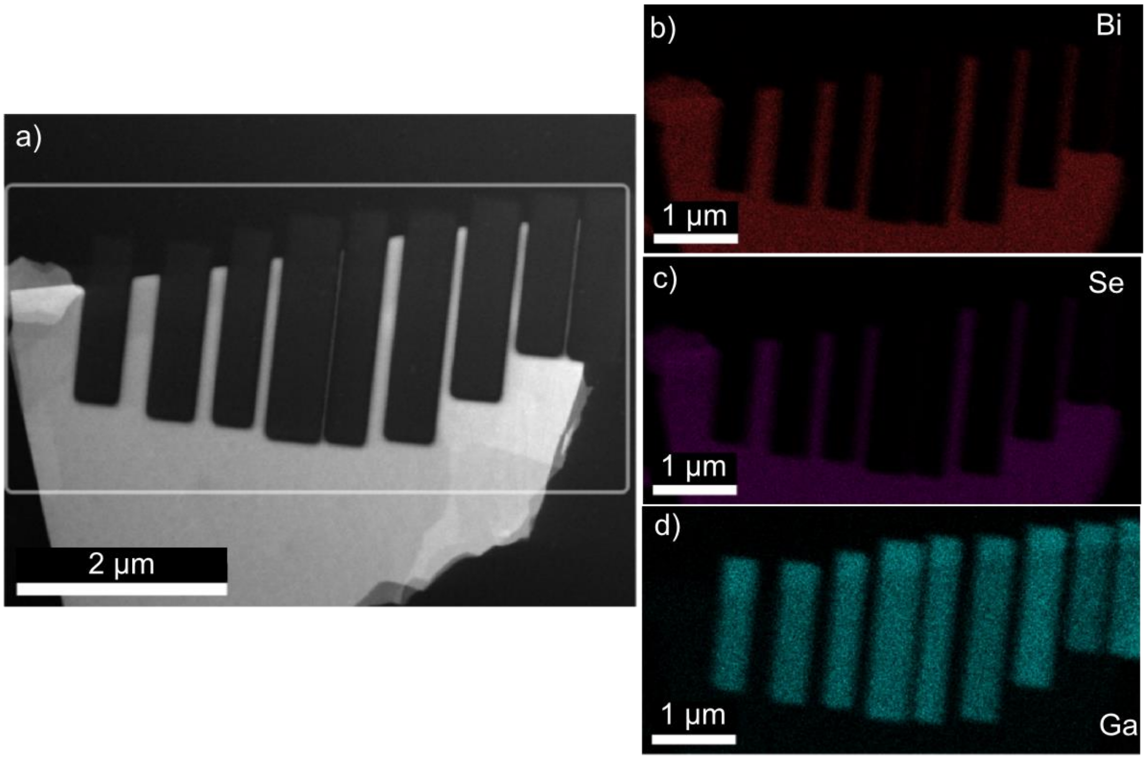

Figure SI 3.1. EDS images of Bi_2Se_3 flake milled to form nanowires ranging width from 260 to 26 nm. (a) Counts per second image of flake. Boxed region was region used for elemental mapping. (b) Bi elemental map. (c) Se elemental map. (d) Ga elemental map.

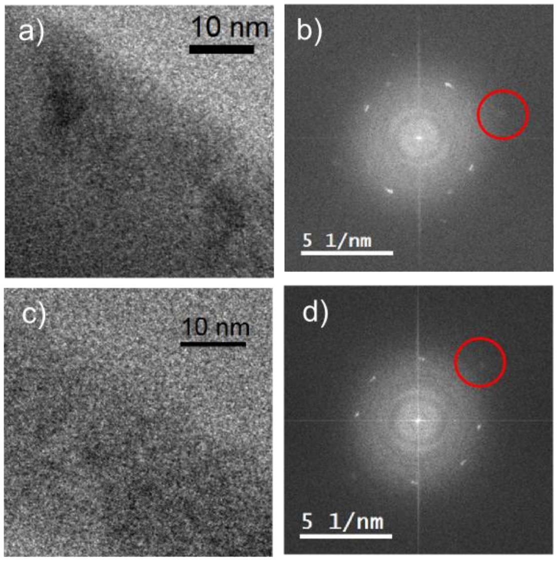

Figure SI 3.2. High resolution and FFT images of wires. (a) HRTEM image of edge of wire 2 from Fig.(a). (b) FFT of (a), encircled in red is point corresponding to Bi_2Se_3 lattice. (c) HRTEM image of wire 4 from Fig. (a). (b) FFT of (c), encircled in red is point corresponding to Bi_2Se_3 lattice.

Section 4: Diagram of sample position in relation to TEM beam

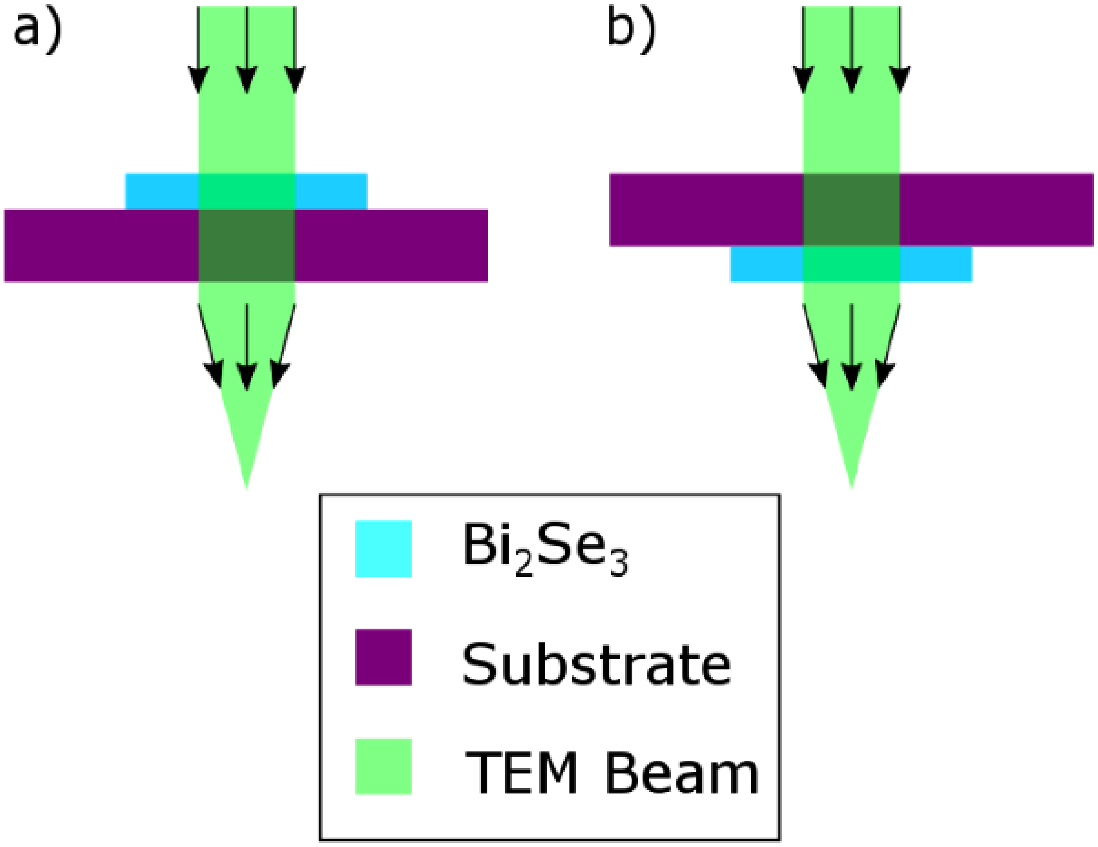

Figure SI 4.1. Diagrams of relation of sample to beam used for TEM measurement.

(a) Sample in "right-side up" position. (b) Sample in "upside down" position.

Section 5: SAED of silicon nitride membrane

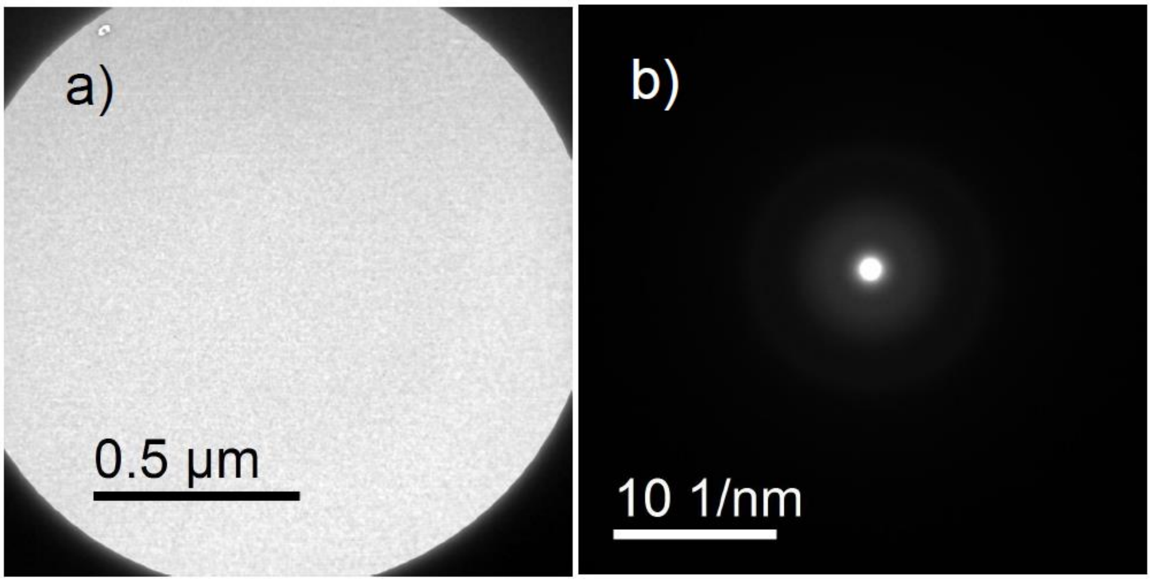

Figure SI 5.1. Selected area and diffraction of silicon nitride membrane. (a) Selected area of silicon nitride membrane. (b) Diffraction image of area of (a).
